# High prevalence and multidrug resistance of Methicillin-resistant *Staphylococcus aureus* at a provincial referral hospital in Zambia: A 5-year retrospective study

**DOI:** 10.1371/journal.pgph.0007348

**Published:** 2026-09-24

**Authors:** Mavis S. Nyakayoya, Dennis Chisala, Kennedy Lubundi, Hariet Milambo, Masiliso A. Liamba, Kesaobaka Batisani, David Chisompola

**Affiliations:** 1 Institute of Basic and Biomedical Sciences, Levy Mwanawasa Medical University, Lusaka, Zambia; 2 Laboratory Department, Afromed Academy of Medical Research and Innovation, Lusaka, Zambia; 3 Microbiology Department, Mansa General Hospital, Mansa, Luapula Province, Zambia; 4 School of Medicine and Health Sciences, University of Lusaka, Lusaka Province, Zambia; 5 Pathology Laboratory Department, Arthur Davison Children’s Hospital, Ndola, Zambia; 6 HAND Research Group, School of Medicine and Health Sciences, Mulungushi University, Livingstone, Zambia; 7 Department of Cardiovascular Science and Metabolic Diseases, Livingstone Center for Prevention and Translational Science, Livingstone, Zambia; Center for Infectious Disease Research in Zambia, ZAMBIA

## Abstract

Methicillin-resistant *Staphylococcus aureus* (MRSA) poses challenges in healthcare settings, contributing to antimicrobial resistance and complicating treatment of staphylococcal infections. In resource-limited settings such as Zambia, staphylococcal infections are treated empirically with clindamycin or co-trimoxazole due to limited susceptibility testing. However, MRSA and multidrug resistance are reducing treatment effectiveness. This study aimed to determine the prevalence and antimicrobial susceptibility of MRSA at Mansa General Hospital, Zambia. A retrospective cross-sectional study was conducted on 266 clinical *S. aureus* isolates from 2020-2024. Bacterial identification was performed using microbiological methods (Mannitol Salt Agar, catalase, coagulase, DNase tests). MRSA detection and antimicrobial susceptibility testing (AST) using the Kirby-Bauer disk diffusion- method with cefoxitin (30 µg) as a surrogate marker, interpreted according to CLSI M100 34th Edition guidelines. Multivariable logistic regression identified factors associated with MRSA. Among 266 *S. aureus* isolates, the overall MRSA prevalence was 31.6% (84/266). MRSA demonstrated significantly lower susceptibility than MSSA to non-beta-lactam antibiotics: clindamycin (46.2% vs. 67.7%), erythromycin (50.0% vs. 79.5%), ciprofloxacin (58.2% vs. 91.4%), and tetracycline (58.3% vs. 81.9%). Chloramphenicol (86.5% vs. 100%) and linezolid (81.8% vs. 97.9%) retained activity. MDR prevalence was 28.6% (76/266), with higher MDR (57.1%, 48/84) than MSSA (15.4%, 28/182). Older age was independently associated with MRSA: adults aged 45–59 years (AOR 2.77; 95% CI 1.09-7.01; p = 0.032) and ≥60 years (AOR 4.53; 95% CI 1.45-14.1; p = 0.009) had higher odds compared to children. Sex, patient type and specimen source were not significant predictors. This study highlights the high prevalence of MRSA at Mansa General Hospital, reduced susceptibility to used oral antibiotics and a burden of multidrug resistance. Empirical use of clindamycin and erythromycin for suspected staphylococcal skin and soft tissue infections should be reconsidered and guided by local antimicrobial susceptibility whenever available. Continued local surveillance and multicenter studies are needed to inform empirical treatment guidelines across Zambia.

## Introduction

Methicillin-resistant *Staphylococcus aureus* (MRSA) remains one of the most significant contributors to the global burden of antimicrobial resistance (AMR) [1]. Characterized by the acquisition of the mecA gene, which encodes an altered penicillin-binding protein (PBP2a) with reduced affinity for beta-lactam antibiotics, MRSA exhibits resistance not only to methicillin but often to multiple other antimicrobial classes, severely complicating treatment options [2,3]. The emergence and spread of MRSA are driven by a combination of factors, including overuse and misuse of antibiotics, inadequate infection control practices, and transmission across both healthcare and community settings [4].

Globally, MRSA imposes a substantial clinical and economic burden. It accounts for 20–80% of all nosocomial *S. aureus* infections, leading to increased mortality, prolonged hospital stays, and elevated healthcare costs [5]. In sub-Saharan Africa, reported MRSA prevalence varies widely, ranging from 1.25% to 53.4%, reflecting significant heterogeneity in surveillance capacity, infection control practices, and antimicrobial stewardship across the region [6].

The challenge in sub-Saharan Africa is further compounded by constrained healthcare infrastructure, inconsistent access to reliable microbiological diagnostics, weak antimicrobial stewardship frameworks, and a persistently high burden of infectious diseases [7]. These factors create an environment conducive to the emergence and dissemination of resistant pathogens. Despite this, comprehensive surveillance data on MRSA from the region remain sparse, with most studies concentrated in tertiary urban centers, leaving significant gaps in understanding the epidemiology of MRSA in rural and provincial healthcare settings [8].

Although MRSA is an emerging public health concern in Zambia, epidemiological data on its prevalence remain limited. Available studies indicate substantial disease burden, including carriage rates among healthcare workers. In one study, *Staphylococcus aureus* carriage was reported at 17.1%, of which 25.8% of isolates were methicillin-resistant [9]. Another Zambian study report has documented MRSA proportions ranging from 37% to 43% among *S. aureus* isolates [10]. These findings, though geographically and methodologically variable, suggest that MRSA may be more widespread than currently appreciated, underscoring the need for systematic national surveillance.

In Zambia, as in many sub-Saharan African countries, empirical treatment of suspected staphylococcal infections is typically guided by WHO Essential Medicines List recommendations and national treatment guidelines, which commonly include clindamycin, erythromycin, and trimethoprim-sulfamethoxazole for skin and soft tissue infections (SSTIs) when parenteral therapy is not required [11,12]. However, these empirical recommendations were developed based on high income countries and historical susceptibility data and may no longer be appropriate in settings with increasing MRSA prevalence and multidrug resistance. Without routine antimicrobial susceptibility testing, clinicians in hospitals must rely on empirical regimens, making contemporary local resistance data essential for guiding prescribing decisions [13].

This study aimed to determine the prevalence and antimicrobial susceptibility patterns of MRSA among *S. aureus* isolates at Mansa General Hospital from 2020 to 2024. The findings will contribute to the World Health Organization’s Global Antimicrobial Resistance and Use Surveillance System (GLASS) priority pathogen surveillance and provide critical evidence to inform local and national infection prevention and control strategies, antimicrobial stewardship programs, and empirical treatment guidelines in similar low-resource settings.

## Materials and methods

### Ethics statement

This study was conducted in accordance with the Declaration of Helsinki and approved by the Levy Mwanawasa Medical University Research Ethics Committee (LMMU-REC) under reference number LMMU-REC 0000541/24 on October 16, 2024. Further regulatory approval was obtained from the National Health Research Authority (NHRA) of Zambia under reference number NHRAR-R-2193/22/11/2024 on 22 November 2024. Permission to access archived isolates and laboratory records was granted by the Senior Medical Superintendent and Hospital Management of Mansa General Hospital. The study was conducted at a single site (Mansa General Hospital), and all study activities were completed within the approved ethical clearance period; therefore, no extension of ethical approval was required. As this was a retrospective study using archived bacterial isolates and de-identified laboratory data, the requirement for individual patient informed consent was waived by the LMMU-REC and NHRA. To ensure patient confidentiality, all data were fully anonymized prior to analysis. Each isolate was assigned a unique study code, and all patient identifiers (names, hospital numbers, addresses) were removed from the database. The anonymized data were stored on a password-protected computer accessible only to the research team. No patient-identifiable information is presented in this manuscript or will be included in any subsequent publications.

### Study design, population and setting

A retrospective, cross-sectional laboratory-based study was conducted at Mansa General Hospital (MGH) from December 03, 2024, to March 30, 2025. Data were retrieved from medical records for participants from January 2020 to November 2024. MGH is a 404-bed capacity provincial referral Hospital. It is a regional referral hospital serving Luapula Province, with specialized departments including pediatrics, surgery, obstetrics-gynecology, and intensive care and functions as a key diagnostic and treatment center for the province and surrounding districts. The study was conducted and reported in accordance with the Strengthening the Reporting of Observational Studies in Epidemiology (STROBE) guidelines (S1 STROBE Checklist) [14].

### Eligibility criteria

All archived, non-duplicate *Staphylococcus aureus* isolates recovered from patients of all ages attending Mansa General Hospital between January 2020 and November 2024 were eligible for inclusion in this study. Duplicate isolates obtained from the same patient during a single infection episode were excluded to prevent overrepresentation. Isolates with incomplete essential demographic or laboratory data were excluded from the respective analyses. In addition, isolates that were non-viable upon subculture, contaminated during revival, or had irretrievable AST records were excluded from the final susceptibility analysis.

### Sample size and selection

A total of 478 non-duplicate *Staphylococcus aureus* isolates, recovered from patients of all ages attending Mansa General Hospital between January 2020 and November 2024, were identified from the microbiology laboratory database and considered for inclusion in this study. From this initial pool, isolates were excluded if they were non-viable upon subculture (n = 41), if their revival resulted in a contaminated culture (n = 18), or if essential corresponding demographic or AST records were incomplete or irretrievable (n = 33). After applying these exclusion criteria, a total of 266 isolates with complete laboratory and demographic data constituted the final analytic cohort for this study.

Missing data were handled by complete-case analysis, as the excluded isolates (n = 212) lacked essential demographic or laboratory data. A formal comparison between included and excluded isolates was not feasible because the excluded isolates had incomplete demographic and clinical information, which constituted the basis for their exclusion. Furthermore, no systematic records were available for non-viable or contaminated cultures beyond their laboratory identification numbers. Consequently, a meaningful assessment of potential selection bias through comparison of included and excluded isolates could not be undertaken.

### Laboratory procedures

#### Bacterial identification and revival.

Archived *S. aureus* isolates were stored at -80°C in cryovials containing beads in cryopreservative fluid (Microbank, Pro-Lab Diagnostics, UK). For revival, a single bead was aseptically removed and inoculated onto Mannitol Salt Agar (MSA; Oxoid, UK), then incubated at 37°C for 18–24 hours. Presumptive identification was based on mannitol fermentation (yellow colonies on MSA). Confirmation was performed using standard phenotypic methods [15]: catalase test (3% hydrogen peroxide), tube coagulase test using rabbit plasma (Becton Dickinson, USA) with observation for clot formation at 4 hours and 24 hours, and DNase test on DNase agar (Oxoid, UK) with 1N HCl flooding to detect clear zones. All tests were performed according to standard operating procedures. Molecular confirmation (e.g., MALDI-TOF or PCR) was not available at the study site.

#### MRSA detection.

Methicillin resistance was detected using the cefoxitin disk diffusion method (30 µg) on Mueller-Hinton Agar (MHA; Oxoid, UK) according to Clinical and Laboratory Standards Institute (CLSI) guidelines [16]. Bacterial suspensions were prepared in sterile saline to a density equivalent to a 0.5 McFarland standard and inoculated onto MHA within 15 minutes. Cefoxitin disks were applied, and plates were incubated at 35°C ± 2°C for 16–18 hours. Inhibition zone diameters were measured and interpreted according to CLSI M100 34th Edition [16]; zones ≤21 mm were classified as methicillin-resistant (MRSA), and zones ≥22 mm as methicillin-susceptible (MSSA).

#### Antimicrobial susceptibility testing.

Antimicrobial susceptibility testing for non-beta-lactam antibiotics was performed using the Kirby-Bauer disk diffusion method on MHA [17]. The following antibiotic disks (Oxoid, UK) were tested based on availability at the time of original isolation: penicillin G (10 units), erythromycin (15 µg), ciprofloxacin (5 µg), clindamycin (2 µg), gentamicin (10 µg), chloramphenicol (30 µg), tetracycline (30 µg), doxycycline (30 µg), levofloxacin (5 µg), trimethoprim-sulfamethoxazole (1.25/23.75 µg), and linezolid (30 µg). Of critical importance, inducible clindamycin resistance (D-zone test) was not performed; therefore, isolates showing erythromycin resistance and clindamycin susceptibility by disk diffusion may include strains with inducible resistance. As a result, the reported clindamycin susceptibility rates are likely to be overestimated, and this represents a significant study limitation.

The specific panel of antibiotics tested was not uniform for all isolates, as testing was based on the hospital’s standard antibiotic panel and disk availability at the time of original isolation between 2020 and 2024. Consequently, the number of isolates tested (n) varies by antibiotic, with smaller sample sizes for certain agents (e.g., doxycycline, levofloxacin, linezolid). This variation is indicated in the results tables, and findings for agents with low numbers tested should be interpreted with caution.

#### Quality control.

*S. aureus* ATCC 25923 was used as the quality control strain for all disk diffusion tests throughout the study period. Results were accepted only when control zones fell within CLSI-specified ranges [17].

#### Definitions.

Multidrug resistance (MDR) was defined as acquired non-susceptibility to at least one agent in three or more antimicrobial categories, according to standardized international criteria [18].

### Data analysis

Data were entered into Microsoft Excel and exported to Stata version 15 (StataCorp LP, College Station, Texas, USA) for statistical analysis. Descriptive statistics were presented as frequencies and percentages for all categorical variables. The prevalence of MRSA was calculated as the proportion of cefoxitin-resistant isolates among all confirmed S. aureus isolates. For comparison of categorical variables between MRSA and MSSA groups, the Chi-square test (χ^2^) was used when expected cell frequencies were ≥5. Fisher’s exact test was applied when expected cell frequencies were <5, particularly for variables with small subgroup sizes (e.g., specimen types other than pus, certain age strata in cross-tabulations). To identify factors independently associated with MRSA, all variables (age group, sex, patient type, and specimen source) were entered into a multivariable logistic regression model using a forced-entry (enter) method. This approach was chosen a priori based on clinical and epidemiological relevance, rather than using stepwise selection, to avoid capitalizing on chance associations. Model fit was assessed using the Hosmer-Lemeshow goodness-of-fit test, and multicollinearity among predictor variables was evaluated using variance inflation factors (VIF), with a threshold of >10 considered indicative of problematic collinearity. No evidence of poor fit (Hosmer-Lemeshow χ² = 0.47, p = 0.791) or substantial multicollinearity (mean VIF = 1.17; all individual VIF < 2.0) was detected. The “urine” specimen category was excluded from the regression model due to quasi-complete separation (zero cell counts), which prevents reliable maximum likelihood estimation. Results are presented as crude odds ratios (OR) and adjusted odds ratios (AOR) with 95% confidence intervals (95% CI). Statistical significance was set at p < 0.05 for all analyses.

## Results

### Demographic and clinical characteristics

The demographic and clinical profile of the 266 participants is summarized in Table 1. The age distribution was bimodal, with the highest burden of infection observed in children (0–10 years, 30.1%) and adults (25–44 years, 31.9%), who together accounted for over two-thirds of cases. Infections showed a male predominance (56.8%). The larger proportion of the isolates were cultured from pus specimens (94.4%), indicative of skin and soft tissue infections (SSTIs) as the primary clinical presentation. The source of infection was nearly evenly distributed between outpatient (50.4%) and inpatient (49.6%) settings.

**Table 1:** Demographic and clinical characteristics of *Staphylococcus aureus* isolates.

Characteristic	Category	Frequency (%)
**Age Group**		
	*Children (0–10 yrs)*	80 (30.1)
	*Adolescents (11–17 yrs)*	29 (10.9)
	*Young adults (18–24 yrs)*	25 (9.4)
	*Adults (25–44 yrs)*	85 (31.9)
	*Old adults (45–59 yrs)*	29 (10.9)
	*Senior adults (≥60 yrs)*	18 (6.8)
**Sex**		
	*Female*	115 (43.2)
	*Male*	151 (56.8)
**Specimen Type**		
	*Pus*	251 (94.4)
	*Blood*	7 (2.6)
	*Urine*	1 (0.4)
	*Other (Sputum and body fluids)*	7 (2.6)
**Patient Type**		
	*Outpatient*	134 (50.4)
	*Inpatient*	132 (49.6)

### Antimicrobial susceptibility patterns

The antimicrobial susceptibility profiles of 266 S. aureus isolates (84 MRSA, 31.6%; 182 MSSA, 68.4%) are presented in Table 2. As expected, all MRSA isolates were resistant to cefoxitin, while all MSSA were susceptible. MRSA demonstrated significantly lower susceptibility than MSSA to key oral antibiotics including clindamycin (46.2% vs. 67.7%; p = 0.006), erythromycin (50.0% vs. 79.5%; p < 0.001), ciprofloxacin (58.2% vs. 91.4%; p < 0.001), and tetracycline (58.3% vs. 81.9%; p = 0.004). Gentamicin susceptibility was also significantly lower in MRSA (76.9% vs. 96.2%; p < 0.001). Chloramphenicol and linezolid retained good activity against both groups, though MRSA showed significantly reduced susceptibility to linezolid (81.8% vs. 97.9%; p = 0.033) and chloramphenicol (86.5% vs. 100%; p < 0.001). Susceptibility to trimethoprim-sulfamethoxazole (61.8% vs. 76.2%; p = 0.114) and doxycycline (72.7% vs. 90.6%; p = 0.577) did not differ significantly between MRSA and MSSA, though the latter may be underpowered due to small sample size. These findings confirm that MRSA in this setting exhibits significantly reduced susceptibility to multiple oral antibiotic classes compared to MSSA, substantially narrowing therapeutic options (Table 2).

**Table 2:** Antimicrobial susceptibility patterns of Methicillin-Resistant *Staphylococcus aureus.*

Antibiotic Name	Isolates Tested (n)	MRSA = 84 (31.6)		MSSA = 182 (68.4)		p-value
		Sensitive n (%)	Resistant n (%)	Sensitive n (%)	Resistant n (%)	
**Cefoxitin (30 µg)**	266	0 (0)	84 (100)	182 (100)	0 (0)	**<0.001**
**Penicillin G (10 U)**	214	2 (3.0)	64 (97.0)	11 (7.4)	137 (92.6)	0.353
**Clindamycin (2 µg)**	179	24 (46.2)	28 (53.8)	86 (67.7)	41 (32.3)	**0.006**
**Erythromycin (15 µg)**	243	36 (50.0)	36 (50.0)	136 (79.5)	35 (20.5)	**<0.001**
**Gentamicin (10 µg)**	185	40 (76.9)	12 (23.1)	128 (96.2)	5 (3.8)	**<0.001**
**Doxycycline (30 µg)**	43	8 (72.7)	3 (27.3)	29 (90.6)	3 (9.4)	0.577
**Tetracycline (30 µg)**	141	21 (58.3)	15 (41.7)	86 (81.9)	19 (18.1)	**0.004**
**Ciprofloxacin (5 µg)**	183	32 (58.2)	23 (41.8)	117 (91.4)	11 (8.6)	**<0.001**
**Levofloxacin (5 µg)**	53	14 (73.7)	5 (26.3)	33 (97.1)	1 (2.9)	**0.018**
**Chloramphenicol (30 µg)**	169	45 (86.5)	7 (13.5)	117 (100)	0 (0)	**<0.001**
**Trimethoprim–Sulfamethoxazole** **(1.25/23.75 µg)**	118	21 (61.8)	13 (38.2)	64 (76.2)	20 (23.8)	0.114
**Linezolid (30 µg)**	69	18 (81.8)	4 (18.2)	46 (97.9)	1 (2.1)	**0.033**

The total tested (n) varies by antibiotic because the susceptibility panel was not uniform for all isolates; antibiotic testing was based on availability of microbial agents and laboratory protocols at the time of isolation, leading to unequal sample sizes across agents. p-values were calculated using Chi-square or Fisher’s exact test comparing susceptibility rates between MRSA and MSSA. Bold indicates statistical significance (p < 0.05).

### Factors associated with MRSA and MSSA among *S. aureus* isolates

In our analysis of factors associated with MRSA, we first conducted univariable screening and then constructed a multivariable logistic regression model to adjust for potential confounders. The findings were largely consistent across both approaches, with age remaining the sole, robust predictor of MRSA isolation.

In the unadjusted analysis, we observed a clear, progressive age-related risk. Compared to the reference group of children (0–10 years), senior adults aged ≥ 60 had significantly higher odds of MRSA (OR 3.97; 95% CI 1.39-11.3; p = 0.010). To identify factors independently associated with MRSA, all variables (age group, sex, patient type, and specimen source) were entered into a multivariable logistic regression model using a forced-entry method. After adjusting for all other variables in the model, these associations not only persisted but strengthened. The risk for adults aged 45–59 increased (AOR 2.77; 95% CI 1.09-7.01; p = 0.032), and for those 60 years and older, the adjusted odds were more than four-and-a-half times that of children (AOR 4.53; 95% CI 1.45-14.1; p = 0.009) (Table 3). This dose-response relationship by age is a compelling signal that warrants attention in risk stratification. However, none of the other variables examined such as sex, patient type, or specimen source, emerged as significant determinants of MRSA in either the crude or adjusted analyses (all p > 0.05) (Table 3).

**Table 3:** Multivariable logistic regression analysis for factors associated with MRSA among *S. aureus* isolates.

Factor	OR 95% CI	p-value	AOR 95% CI	p-value
**Age group (years)**				
*Children (0–10)*	Ref		Ref	
*Adolescents (11–17)*	1.44 (0.56, 3.70)	0.443	1.47 (0.57, 3.82)	0.428
*Young adults (18–24)*	1.81 (0.69, 4.74)	0.230	1.88 (0.70, 5.04)	0.208
*Adults (25–44)*	1.34 (0.66, 2.68)	0.412	1.52 (0.74, 3.12)	0.254
*Older adults (45–59)*	2.27 (0.92, 5.58)	0.075	2.77 (1.09, 7.01)	**0.032**
*Senior adults (≥60)*	3.97 (1.39, 11.3)	**0.010**	4.53 (1.45, 14.1)	**0.009**
**Sex**				
*Male*	Ref		Ref	
*Female*	1.04 (0.62, 1.76)	0.855	1.14 (0.66, 1.96)	0.638
**Patient Type**				
*Inpatient*	Ref		Ref	
*Outpatient*	0.77 (0.46, 1.29)	0.332	0.72 (0.41, 1.26)	0.256
**Specimen Type**				
*Pus*	Ref		Ref	
*Blood*	2.95 (0.64, 13.5)	0.162	1.86 (0.36, 9.52)	0.454
*Other (Sputum and body fluids)*	0.36 (0.04, 3.12)	0.361	0.28 (0.03, 2.52)	0.260

The “Urine” category was dropped from the logistic regression analysis due to quasi-complete separation (or zero cell counts), where the cross-tabulation of urine specimens with the MRSA outcome resulted in empty cells. Under such conditions, maximum likelihood estimates cannot be reliably calculated, and any odds ratio generated would be invalid.

### Multidrug-resistant pattern of *S. aureus* isolates

Analysis of the 266 *S. aureus* isolates revealed a stark contrast in the multidrug-resistant (MDR) patterns between Methicillin-Resistant (MRSA) and Methicillin-Susceptible (MSSA) strains. Overall, 28.6% (76/266) of all isolates were classified as MDR, defined as resistance to three or more antimicrobial classes. However, this burden was disproportionately carried by the MRSA population; among the 84 MRSA isolates, the majority (57.1%, n = 48) were MDR, with a significant portion demonstrating resistance to five or more drug classes (22.6%). In contrast, the 182 MSSA isolates exhibited a much lower rate of multidrug resistance at just 15.4% (28/182), with the vast majority showing resistance to only one or two classes. These findings highlight that MRSA is not only defined by its beta-lactam resistance but is also far more likely to possess additional resistance, presenting a greater therapeutic challenge compared to MSSA (Table 4).

**Table 4:** Multidrug-resistant pattern of *S. aureus* isolates.

*S. aureus* (n = 266)	Resistance patterns						
	R0	R1	R2	R3	R4	R5	MDR (%)
MRSA = 84	4 (4.8)	24 (28.6)	8 (9.5)	13 (15.5)	16 (19.0)	19 (22.6)	48 (57.1)
MSSA = 182	26 (14.3)	88 (48.4)	40 (22.0)	18 (9.9)	4 (2.2)	6 (3.3)	28 (15.4)
Total = 266	30 (11.3)	112 (42.1)	48 (18.0)	31 (11.7)	20 (7.5)	25 (9.4)	76 (28.6)

MSSA refers to methicillin-susceptible *Staphylococcus aureus*, while MRSA denotes methicillin-resistant *Staphylococcus aureus*. R0: no resistance; R1: resistant to one antibiotic; R2: resistant to two antibiotics; R3: resistant to three antibiotics; R4: resistant to four antibiotics; R5: resistant to five or more antibiotics.

## Discussion

This study provides important contemporary data on *Staphylococcus aureus* infections at Mansa General Hospital, revealing three important findings: a high MRSA prevalence of 31.6%, significantly reduced susceptibility of MRSA to multiple oral antibiotics including clindamycin (46.2%) and erythromycin (50.0%), and a strong association between MRSA and multidrug resistance (57.1% of MRSA isolates were MDR). These findings have immediate implications for empirical treatment guidelines, infection control practices, and antimicrobial stewardship programs in this and similar resource-limited settings.

The MRSA prevalence falls within the upper range reported from sub-Saharan Africa (1.25-53.4%) [6,19]. Beyond methicillin resistance, MRSA revealed significantly lower susceptibility than MSSA to key oral antibiotics: clindamycin, erythromycin, ciprofloxacin, and tetracycline. These findings indicate that clindamycin and erythromycin may no longer be reliable empirical options for suspected staphylococcal infections in this setting. Moreover, several agents showed good activity against MRSA: trimethoprim-sulfamethoxazole, doxycycline, chloramphenicol, and linezolid. However, it is critical to emphasize that the estimates for doxycycline, levofloxacin, and linezolid are based on very small subsamples, and these point estimates are too imprecise to guide clinical practice. Trimethoprim-sulfamethoxazole and doxycycline remain viable oral options for uncomplicated skin and soft tissue infections (SSTIs), consistent with IDSA guidelines [20,21]. However, the wide confidence interval for doxycycline renders it statistically unreliable for clinical decision-making. This estimate should be interpreted with caution. Linezolid, despite showing reasonable activity in our limited sample, should be reserved for severe infections due to cost and toxicity concerns, and its apparent high susceptibility should not be interpreted as a definitive population estimate given the very small number of isolates tested.

MRSA isolates demonstrated significantly lower susceptibility than MSSA to nearly all tested non-beta-lactam antibiotics. The most pronounced differences were observed for ciprofloxacin, erythromycin, and tetracycline [22]. These findings indicate that commonly used oral antibiotics can no longer be relied upon for empirical therapy of suspected staphylococcal infections. In contrast, the observed MRSA susceptibility to clindamycin requires not just cautious interpretation, but outright acknowledgment of a critical study limitation. Because D-zone testing for inducible resistance was not performed, isolates with inducible macrolide-lincosamide-streptogramin B (MLSb) resistance would have been misclassified as clindamycin-susceptible [23]. Therefore, the clindamycin susceptibility rate is likely a significant overestimate of true clindamycin activity. Consequently, based on these data, clindamycin cannot be recommended as first-line empirical therapy for suspected MRSA infections in this setting without confirmatory D-zone testing.

A key finding is the association between MRSA and multidrug resistance. Among MRSA isolates, nearly half met MDR criteria (resistance to ≥3 classes) compared to MSSA. Similar with what has been reported in other studies where MRSA MDR is higher than in MSSA [22,24,25]. Nearly a quarter of MRSA exhibited resistance to five or more antibiotic classes, representing extensively resistant phenotypes that pose significant therapeutic challenges. This confirms that MRSA in our setting is not merely beta-lactam resistant but carries a broad resistance arsenal, reinforcing the need for routine susceptibility testing to guide therapy.

Increasing age emerged as an independent predictor of MRSA, highlighting a clear dose-response relationship. Compared to children (0–12 years), adults aged 36–59 years had nearly three-fold higher odds and those ≥60 years had more than four-fold higher odds. This likely reflects cumulative healthcare exposure, age-related comorbidities, and prior antibiotic use, all these are established risk factors for MRSA colonization and infection [26]. Interestingly, patient type (inpatient vs. outpatient) was not significantly associated with MRSA in multivariable logistic regression model. While this finding could be interpreted as suggestive of a blurring of traditional healthcare-associated and community-associated MRSA distinctions, such an interpretation must be made with considerable caution. Our study did not collect data on prior healthcare exposures, antibiotic use histories, comorbidities, or molecular strain types (e.g., SCCmec typing), all of which are essential for definitively classifying MRSA as healthcare-associated or community-associated. It is plausible that older adults presenting with MRSA infections in the outpatient department have acquired the organism through prior healthcare visits, a possibility we could not assess, or that risk factors for MRSA (e.g., chronic diseases, frequent healthcare contacts) accumulate with age, irrespective of current admission status. Without molecular characterization and detailed exposure histories, we cannot determine whether these infections are truly community-associated or reflect unrecognized healthcare exposure.

These findings have several practical implications: 1) Empirical therapy for SSTIs should be reevaluated clindamycin and erythromycin are no longer reliable; TMP-SMX or doxycycline are more rational oral options; 2) Routine susceptibility testing is essential, particularly for severe infections; 3) Older adults should be prioritized for enhanced infection control measures; 4) The blurring of community-healthcare boundaries calls for integrated infection control strategies spanning both settings.

### Limitations

This study has several limitations to acknowledge. First, the final sample of 266 isolates represents a convenience sample of viable isolates with complete records from the 478 initially identified. The exclusion of 212 isolates (44.4%) due to non-viability, contamination, or incomplete data introduces a potential risk of selection bias. Consequently, it was not possible to determine whether the included isolates were fully representative of the original isolate pool or whether systematic differences existed between included and excluded cases. The findings should be interpreted with appropriate caution when considering their generalizability. Second, the non-uniform antibiotic testing panels resulted in small sample sizes for several clinically important agents (e.g., linezolid, doxycycline), limiting the precision and generalizability of those specific susceptibility estimates. Third, the failure to perform D-zone testing for inducible clindamycin resistance means that clindamycin susceptibility is almost certainly overestimated, and these results should not guide therapy. Fourth, MRSA identification in this study was based on cefoxitin disk diffusion, which is the CLSI-recommended phenotypic surrogate for mecA-mediated methicillin resistance. However, molecular confirmation using detection of mecA or mecC genes was not available. Although cefoxitin disk diffusion has high diagnostic accuracy, the absence of molecular confirmation remains a limitation and may have resulted in a small degree of isolate misclassification. Fifth, as a retrospective single-center study conducted at a provincial referral hospital, the findings may not be generalizable to other regions of Zambia or to primary healthcare facilities. Finally, clinical outcome data were unavailable, preventing correlation of resistance patterns with treatment outcomes

## Conclusion

This study reveals a high MRSA prevalence at Mansa General Hospital, with MRSA isolates highlighting significantly reduced susceptibility to multiple oral antibiotics and a strong association with multidrug resistance. Age was significantly associated with MRSA, with older adults at substantially higher risk, and the lack of association with inpatient status suggests that MRSA transmission may extend beyond traditional healthcare boundaries. These findings suggest that the routine empirical use of clindamycin and erythromycin for suspected staphylococcal skin and soft tissue infections should be reconsidered in this setting, as the observed susceptibility rates are substantially lower than would be acceptable for empirical therapy. However, these agents may still have a role in individual patients where susceptibility is confirmed by testing. Furthermore, Trimethoprim-sulfamethoxazole may represent a more reliable empirical oral option based on our data, though routine susceptibility testing should guide definitive therapy whenever possible. Linezolid showed activity against most MRSA isolates in this limited sample; however, it should be reserved for severe, confirmed infections given its WHO Reserve status, toxicity, cost, and limited availability, and avoided for empirical treatment of uncomplicated cases. Routine susceptibility testing, continuous local surveillance, and molecular characterization of circulating strains are needed to monitor resistance trends, guide therapeutic decisions, and inform targeted public health interventions in Zambia and similar settings.

## Supporting information

S1 STROBE Checklist STROBE Checklist. (DOCX)

S1 Data Data. (XLSX)

## Abbreviations

AMR: antimicrobial resistance
AST: antimicrobial susceptibility testing
ATCC: American type culture collection
CLSI: clinical and laboratory standards institute
DNA: deoxyribonucleic acid
GLASS: global antimicrobial resistance and use surveillance system
IDSA: infectious diseases society of America
MALDI-TOF: matrix-assisted laser desorption/ionization-time of flight
MDR: multidrug resistance/multidrug-resistant
MHA: mueller-Hinton agar
MLSb: macrolide-lincosamide-streptogramin B
MRSA: methicillin-resistant *Staphylococcus aureus*
MSA: mannitol salt agar
MSSA: methicillin-susceptible *Staphylococcus aureus*
NHRA: national health research authority
PBP2a: penicillin-binding protein 2a
PCR: polymerase chain reaction
SCCmec: staphylococcal cassette chromosome mec
SSTI: skin and soft tissue infection
TMP-SMX: trimethoprim-sulfamethoxazole
WHO: World Health Organization
