# Supplementary material for: High prevalence and multidrug resistance of Methicillin-resistant Staphylococcus aureus at a provincial referral hospital in Zambia: A 5-year retrospective study
Source: PLOS Glob Public Health. 2026 Sep 24;6(9):e0007348. doi: 10.1371/journal.pgph.0007348 (PMC13606941; doi:10.1371/journal.pgph.0007348)
Supplement: Supplementary file 2 [file pgph.0007348.s003.docx]

**Response to comments**

We thank the reviewers and editors for their time to review this work. We have revised the manuscript addressing all the comments raised and have uploaded the tracked and clean copy of the manuscript. Find the response below in blue fonts.

1. We note that your Data Availability Statement is currently as follows: The raw data underlying the results presented in the study have been uploaded as supporting information S2.
    
    Please confirm at this time whether or not your submission contains all raw data required to replicate the results of your study. Authors must share the “minimal data set” for their submission. PLOS defines the minimal data set to consist of the data required to replicate all study findings reported in the article, as well as related metadata and methods (<https://journals.plos.org/plosone/s/data-availability#loc-minimal-data-set-definition>).
    
    For example, authors should submit the following data:
    
    - The values behind the means, standard deviations and other measures reported;
    - The values used to build graphs;
    - The points extracted from images for analysis.
    
    Authors do not need to submit their entire data set if only a portion of the data was used in the reported study.
    
    If your submission does not contain these data, please either upload them as Supporting Information files or deposit them to a stable, public repository and provide us with the relevant URLs, DOIs, or accession numbers. For a list of recommended repositories, please see <https://journals.plos.org/plosone/s/recommended-repositories>.
    
    If there are ethical or legal restrictions on sharing a de-identified data set, please explain them in detail (e.g., data contain potentially sensitive information, data are owned by a third-party organization, etc.) and who has imposed them (e.g., an ethics committee). Please also provide contact information for a data access committee, ethics committee, or other institutional body to which data requests may be sent. If data are owned by a third party, please indicate how others may request data access.

**Response**

We thank the reviewer for this important clarification. We already uploaded the raw data as supplementary file S2.

2. We do not publish any copyright or trademark symbols that usually accompany proprietary names, eg (R), (C), or TM  (e.g. next to drug or reagent names). Please remove all instances of trademark/copyright symbols throughout the text, including ATCC® and Microbank™.

**Response**

We thank the reviewer for this important observation. We have removed all copyright/trademark symbols.

3. Please ensure that your Ethics Statement is available in its entirety at the beginning of your Methods section, under a subheading 'Ethics Statement'.

**Response**

We thank the reviewer for this important observation. We have revised the heading of the ethics statement in the methods section and have move it to the beginning of the methods section.

**Review Comments to the Author**

Please use the space provided to explain your answers to the questions above. You may also include additional comments for the author, including concerns about dual publication, research ethics, or publication ethics. (Please upload your review as an attachment if it exceeds 20,000 characters)

**Reviewer #1:** This manuscript addresses an important public health issue by describing the prevalence, antimicrobial susceptibility patterns, and multidrug resistance characteristics of Methicillin-Resistant Staphylococcus aureus (MRSA) at a provincial referral hospital in Zambia. The study contributes valuable data from a setting where antimicrobial resistance surveillance remains limited and therefore has potential relevance for antimicrobial stewardship, infection prevention and control, and national surveillance efforts.

Overall, the study is methodologically sound, ethically conducted, and generally well reported. The retrospective cross-sectional design is appropriate for the study objectives, laboratory procedures are described in sufficient detail, and antimicrobial susceptibility testing was performed according to CLSI guidelines. Ethical approval and data confidentiality procedures are adequately documented. The statistical analyses are generally appropriate, including the use of chi-square/Fisher's exact tests and multivariable logistic regression to identify factors associated with MRSA.

The results support the principal conclusions that MRSA prevalence is high in this setting, that MRSA isolates exhibit substantially higher multidrug resistance than MSSA isolates, and that increasing age is associated with higher odds of MRSA isolation. The findings provide useful local evidence that may inform clinical practice and antimicrobial stewardship initiatives.

However, several issues should be addressed during revision:
The exclusion of 212 of the 478 initially identified isolates (44.4%) raises concerns regarding potential selection bias. The authors should discuss whether included and excluded isolates differed systematically and, where possible, provide a c5omparison of available characteristics.

**Response**

We agree with the reviewer that the high exclusion rate (44.4%) is a significant limitation that warrants thorough examination. Because of missing key demographic and clinical variables, we were unable to perform comparisons between the included and excluded isolates. We have added this to the methods section and expanded on this limitation.

The antibiotic susceptibility panel was not uniform across all isolates, resulting in substantially smaller sample sizes for some antibiotics, including doxycycline, levofloxacin, and linezolid. The authors appropriately acknowledge this limitation, but the interpretation of susceptibility estimates for these agents should be further tempered.

**Response**

We thank the reviewer for this insightful comment. We have revised the discussion section accordingly. We have added a caution to the interpretation of these antibiotics (doxycycline, levofloxacin, and linezolid).

The absence of D-zone testing represents an important limitation. Because inducible clindamycin resistance was not assessed, clindamycin susceptibility may be overestimated. Consequently, recommendations regarding clindamycin use should be presented more cautiously.
**Response**

We thank the reviewer for this important observation. We stated the absence of D-zone, we have revised to make it clearer and have rephrased the recommendations.

Molecular confirmation of MRSA and molecular characterization of isolates were not performed. While understandable in a resource-limited setting, the absence of molecular testing should be emphasized when interpreting epidemiological inferences.

**Response**

We thank the reviewer for this important observation. We have revised the discussion section and have stated the absence of molecular characterisation.

The statement suggesting a “blurring of community-healthcare boundaries” should be framed more cautiously because the study did not assess prior healthcare exposure, antibiotic use, comorbidities, or molecular strain types.
**Response**

We thank the reviewer for this important observation. We have revised the discussion section and have reframed with a cautiously interpretation statement.

The recommendation that empirical use of clindamycin and erythromycin should be “abandoned” may be stronger than warranted by the available data. A more balanced recommendation would be to reconsider their routine empirical use pending further surveillance and susceptibility testing.

**Response**

We thank the reviewer for these insightful comments given. We have revised accordingly in both the main text conclusion section as well as abstract conclusion section.

The manuscript would benefit from minor improvements in statistical reporting, including consideration of logistic regression model diagnostics and clarification of missing data handling.
**Response**

We thank the reviewer for this important observation. The manuscript already contains logistic regression analysis under the section on factors associated with MRSA. We have revised the heading to include Logistic regression analysis to make the statement clear. We have also expanded on the logistic regression analyses performed.

Minor grammatical, stylistic, and typographical errors should be corrected. Examples include inconsistent capitalization, minor punctuation issues, and a spelling error in the reference list (“Susuceptibility” should read “Susceptibility”).
**Response**

We thank the reviewer for this important observation. We have corrected the grammatical and typographic errors.

Regarding data availability, the manuscript states that the raw data underlying the findings have been provided as Supporting Information (S2). Compliance with PLOS data-sharing requirements should be confirmed by ensuring that the complete anonymized dataset and sufficient variable descriptions are available to permit reproduction of the analyses.

**Response**
We thank the reviewer for raising this important point regarding data availability and compliance with PLOS data-sharing requirements. We complied with PLOS data-sharing requirement. We have revised the raw data to ensure it complies.

I found no major concerns regarding research ethics, publication ethics, or dual publication. Ethical approval, waiver of informed consent, and measures to protect patient confidentiality were appropriately described.
**Response**

We thank the reviewer for the feedback given.

In summary, this is a valuable contribution to the antimicrobial resistance literature from Zambia and an underrepresented healthcare setting. Subject to satisfactory revision of the points outlined above, the manuscript would be suitable for publication.

**Response**

We thank the reviewer for the thorough review of our work and the insightful comments provided meant to improve our work.

**Reviewer #2:** I want to commend the authors for this commendable work. Overall, the manuscript is well-written and speaks to a very pertinent and important public health issue in the context of Zambia and Africa. Furthermore, the issue of antimicrobial resistance is an important subject matter within the clinical and public health response. Once again, I commend the team for a job well done. However, there are minor corrections that the authors need to look into.
1. To begin with, the background in the abstract refers to MRSA being a concern for infection control in low-income settings. This study did not focus on infection control; therefore, this aspect may not warrant inclusion. This was a laboratory-based retrospective study with no focus on infection control, so this may need to be removed from the abstract. Additionally, the issue of MRSA is not limited to low-incomesettings. This is a global issue, so the authors should consider framing it in that context.

**Response**

We thank the reviewer for this important observation and agree with both points raised. We have revised and removed both points. Additionally, we have corrected from limited resource centre to worldwide.

2. In the background, it would help put the study into context if the authors state what is empirically prescribed in the case of MRSA. I think this is missing in both the abstract and the main document. Including this will strengthen the current draft's argument regarding why exploring resistance to MRSA matters in the context of empirical prescription

**Response**

We thank the reviewer for this important suggestion. We have revised the background section for both the main text as well as the abstract and expanded on empirical prescriptions.

3. In the results section, the age band categories need to be rewritten for clarity, likely as: 0–10, 11–17, 18–24, 25–44, 45–59, and 60 and above.

Response

We thank the reviewer for this important suggestion. We have revised accordingly and have re-performed statistical analysis were applicable to present the findings on the year age band.

4. In line 212 the sentence that includes overwhelming majority, the words overwelming majority does not read well for a scientific paper, consider replacing with more appropriate like a “larger proportion”

**Response**

We thank the reviewer for this important observation. We have revised accordingly.

5. It will be worthwhile to state what constituted others for the specimens explored in the study, this can be put in blackets

**Response**

We thank the reviewer for this important suggestion. We have revised accordingly.

6. Coming to the discussion: The authors have included results in this section. Results should not be repeated in the discussion. Instead, the authors must make inferences from the results. What is their significance? How do the results compare with other studies? They should also discuss the results in terms of their implications within the current clinical context.

**Response**

We thank the reviewer for this important observation. We have revised the discussion section accordingly. We have removed results and have interpreted the results.

7. The public health context should not simply restate the findings; thus, the raw results must be entirely removed from the discussion section of the manuscript.

**Response**

We thank the reviewer for this important observation. We have revised the discussion section accordingly. We have removed raw results.

8. Lastly, I would like to suggest that the authors consider including a recommendation on Linezolid to be prioritised in empirical treatment of MRSA.

Response

We thank the reviewer for this important suggestion. We have revised accordingly.
