# Supplementary material for: High prevalence and multidrug resistance of Methicillin-resistant Staphylococcus aureus at a provincial referral hospital in Zambia: A 5-year retrospective study
Source: PLOS Glob Public Health. 2026 Sep 24;6(9):e0007348. doi: 10.1371/journal.pgph.0007348 (PMC13606941; doi:10.1371/journal.pgph.0007348)
Supplement: Supplementary file 3 [file pgph.0007348.s004.docx]

**Response to Reviewers**

I have only a few minor comments:

The conclusions should be slightly more cautious. Since this is a single-centre retrospective study, recommendations regarding empirical antibiotic use should be framed within the context of the study setting.

**Response**

We thank the reviewer for this valuable suggestion. We have revised the conclusion to adopt more cautious language and explicitly state that recommendations regarding empirical antibiotic therapy apply to the study setting (Mansa General Hospital) and should not be generalized without local surveillance data

Please verify whether Mansa General Hospital should be described as a tertiary hospital or a provincial referral hospital and ensure this is consistent throughout the manuscript.

**Response**

Thank you for identifying this inconsistency. We have reviewed the manuscript and corrected the terminology throughout. Mansa General Hospital is now consistently described as a provincial referral hospital.

It would be useful to briefly acknowledge in the discussion that cefoxitin disk diffusion is a phenotypic surrogate for MRSA detection and that molecular confirmation was not available.

**Response**

We appreciate this suggestion. We have added a statement in the Discussion acknowledging that cefoxitin disk diffusion is a validated phenotypic surrogate for MRSA detection according to CLSI guidelines, while recognizing that molecular confirmation (e.g., mecA/mecC PCR) was not available and may have resulted in some misclassification.

A final proofread is recommended, as there are a few minor grammatical and typographical errors that remain.

**Response**

We thank the reviewer for this suggestion. The manuscript has undergone a thorough proofread, and minor grammatical, typographical, and formatting inconsistencies have been corrected throughout.

We have also corrected the results in the abstract and under table 3 narration to consistently reflect what is in table 3 (multivariable logistic regression).

Overall, this is a well-conducted study that provides useful info]rmation on the burden of MRSA and antimicrobial resistance in Zambia. The findings are relevant to antimicrobial stewardship and infection prevention efforts, particularly in resource-limited settings. Subject to these minor revisions, I believe the manuscript is suitable for publication.

**Response**

We thank the reviewer and editorial team for taking their valuable time to review this manuscript. We believe that the suggested corrections have substantially improved our manuscript.
