# Supplementary material for: High prevalence and multidrug resistance of Methicillin-resistant Staphylococcus aureus at a provincial referral hospital in Zambia: A 5-year retrospective study
Source: PLOS Glob Public Health. 2026 Sep 24;6(9):e0007348. doi: 10.1371/journal.pgph.0007348 (PMC13606941; doi:10.1371/journal.pgph.0007348)
Supplement: Peer review history

# Peer review: Decision Letter 0

**DOI:** 10.1371/journal.pgph.0007348.r001

**Authors:** Monde Muyoyeta

1 Jul 2026

PGPH-D-26-01568

High Prevalence and Multidrug Resistance of Methicillin-Resistant Staphylococcus aureus in a Provincial Referral Hospital in Zambia: A 5-Year Retrospective Study

PLOS Global Public Health

Dear Corresponding Author,

Thank you for submitting your manuscript to PLOS Global Public Health. After careful consideration, we feel that it has merit but does not fully meet PLOS Global Public Health’s publication criteria as it currently stands. Therefore, we invite you to submit a revised version of the manuscript that addresses the points raised during the review process.

Please submit your revised manuscript by Aug 30 2026 11:59PM. If you will need more time than this to complete your revisions, please reply to this message or contact the journal office at globalpubhealth@plos.org. When you're ready to submit your revision, log on to https://www.editorialmanager.com/pgph/ and select the 'Submissions Needing Revision' folder to locate your manuscript file.

Please include the following items when submitting your revised manuscript:

- A letter that responds to each point raised by the editor and reviewer(s). You should upload this letter as a separate file labeled 'Response to Reviewers'.
- A marked-up copy of your manuscript that highlights changes made to the original version. You should upload this as a separate file labeled 'Revised Manuscript with Track Changes'.
- An unmarked version of your revised paper without tracked changes. You should upload this as a separate file labeled 'Manuscript'.

Guidelines for resubmitting your figure files are available below the reviewer comments at the end of this letter.

As the corresponding author, your ORCID iD is verified in the submission system and will appear in the published article. PLOS supports the use of ORCID, and we encourage all coauthors to register for an ORCID iD and use it as well. Please encourage your coauthors to verify their ORCID iD within the submission system before final acceptance, as unverified ORCID iDs will not appear in the published article. *Only*  the individual author can complete the verification step; PLOS staff *cannot*  verify ORCID iDs on behalf of authors.

We look forward to receiving your revised manuscript.

Kind regards,

Monde Muyoyeta, MBCHB, Ph.D

Academic Editor

PLOS Global Public Health

**Journal Requirements:**

1. We note that your Data Availability Statement is currently as follows: The raw data underlying the results presented in the study have been uploaded as supporting information S2.

Please confirm at this time whether or not your submission contains all raw data required to replicate the results of your study. Authors must share the “minimal data set” for their submission. PLOS defines the minimal data set to consist of the data required to replicate all study findings reported in the article, as well as related metadata and methods (https://journals.plos.org/plosone/s/data-availability#loc-minimal-data-set-definition).

For example, authors should submit the following data:

- The values behind the means, standard deviations and other measures reported;

- The values used to build graphs;

- The points extracted from images for analysis.

Authors do not need to submit their entire data set if only a portion of the data was used in the reported study.

If your submission does not contain these data, please either upload them as Supporting Information files or deposit them to a stable, public repository and provide us with the relevant URLs, DOIs, or accession numbers. For a list of recommended repositories, please see https://journals.plos.org/plosone/s/recommended-repositories.

If there are ethical or legal restrictions on sharing a de-identified data set, please explain them in detail (e.g., data contain potentially sensitive information, data are owned by a third-party organization, etc.) and who has imposed them (e.g., an ethics committee). Please also provide contact information for a data access committee, ethics committee, or other institutional body to which data requests may be sent. If data are owned by a third party, please indicate how others may request data access.

2. We do not publish any copyright or trademark symbols that usually accompany proprietary names, eg (R), (C), or TM  (e.g. next to drug or reagent names). Please remove all instances of trademark/copyright symbols throughout the text, including ATCC® and Microbank™.

3. Please ensure that your Ethics Statement is available in its entirety at the beginning of your Methods section, under a subheading 'Ethics Statement'.

If the reviewer comments include a recommendation to cite specific previously published works, please review and evaluate these publications to determine whether they are relevant and should be cited. There is no requirement to cite these works unless the editor has indicated otherwise.

**Additional Editor Comments (if provided):**

[Note: HTML markup is below. Please do not edit.]

Reviewers' comments:

Reviewer's Responses to Questions

**Comments to the Author**

1. Does this manuscript meet PLOS Global Public Health’s publication criteria?

Reviewer #1: Yes

Reviewer #2: Yes

**********

2. Has the statistical analysis been performed appropriately and rigorously?

Reviewer #1: Yes

Reviewer #2: Yes

**********

3. Have the authors made all data underlying the findings in their manuscript fully available (please refer to the Data Availability Statement at the start of the manuscript PDF file)?

The PLOS Data policy

Reviewer #1: Yes

Reviewer #2: No

**********

4. Is the manuscript presented in an intelligible fashion and written in standard English?

Reviewer #1: Yes

Reviewer #2: Yes

**********

**Reviewer #1:**  This manuscript addresses an important public health issue by describing the prevalence, antimicrobial susceptibility patterns, and multidrug resistance characteristics of Methicillin-Resistant Staphylococcus aureus (MRSA) at a provincial referral hospital in Zambia. The study contributes valuable data from a setting where antimicrobial resistance surveillance remains limited and therefore has potential relevance for antimicrobial stewardship, infection prevention and control, and national surveillance efforts.

Overall, the study is methodologically sound, ethically conducted, and generally well reported. The retrospective cross-sectional design is appropriate for the study objectives, laboratory procedures are described in sufficient detail, and antimicrobial susceptibility testing was performed according to CLSI guidelines. Ethical approval and data confidentiality procedures are adequately documented. The statistical analyses are generally appropriate, including the use of chi-square/Fisher's exact tests and multivariable logistic regression to identify factors associated with MRSA.

The results support the principal conclusions that MRSA prevalence is high in this setting, that MRSA isolates exhibit substantially higher multidrug resistance than MSSA isolates, and that increasing age is associated with higher odds of MRSA isolation. The findings provide useful local evidence that may inform clinical practice and antimicrobial stewardship initiatives.

However, several issues should be addressed during revision:

The exclusion of 212 of the 478 initially identified isolates (44.4%) raises concerns regarding potential selection bias. The authors should discuss whether included and excluded isolates differed systematically and, where possible, provide a comparison of available characteristics.

The antibiotic susceptibility panel was not uniform across all isolates, resulting in substantially smaller sample sizes for some antibiotics, including doxycycline, levofloxacin, and linezolid. The authors appropriately acknowledge this limitation, but the interpretation of susceptibility estimates for these agents should be further tempered.

The absence of D-zone testing represents an important limitation. Because inducible clindamycin resistance was not assessed, clindamycin susceptibility may be overestimated. Consequently, recommendations regarding clindamycin use should be presented more cautiously.

Molecular confirmation of MRSA and molecular characterization of isolates were not performed. While understandable in a resource-limited setting, the absence of molecular testing should be emphasized when interpreting epidemiological inferences.

The statement suggesting a “blurring of community-healthcare boundaries” should be framed more cautiously because the study did not assess prior healthcare exposure, antibiotic use, comorbidities, or molecular strain types.

The recommendation that empirical use of clindamycin and erythromycin should be “abandoned” may be stronger than warranted by the available data. A more balanced recommendation would be to reconsider their routine empirical use pending further surveillance and susceptibility testing.

The manuscript would benefit from minor improvements in statistical reporting, including consideration of logistic regression model diagnostics and clarification of missing data handling.

Minor grammatical, stylistic, and typographical errors should be corrected. Examples include inconsistent capitalization, minor punctuation issues, and a spelling error in the reference list (“Susuceptibility” should read “Susceptibility”).

Regarding data availability, the manuscript states that the raw data underlying the findings have been provided as Supporting Information (S2). Compliance with PLOS data-sharing requirements should be confirmed by ensuring that the complete anonymized dataset and sufficient variable descriptions are available to permit reproduction of the analyses.

I found no major concerns regarding research ethics, publication ethics, or dual publication. Ethical approval, waiver of informed consent, and measures to protect patient confidentiality were appropriately described.

In summary, this is a valuable contribution to the antimicrobial resistance literature from Zambia and an underrepresented healthcare setting. Subject to satisfactory revision of the points outlined above, the manuscript would be suitable for publication.

**Reviewer #2:**  I want to commend the authors for this commendable work. Overall, the manuscript is well-written and speaks to a very pertinent and important public health issue in the context of Zambia and Africa. Furthermore, the issue of antimicrobial resistance is an important subject matter within the clinical and public health response. Once again, I commend the team for a job well done. However, there are minor corrections that the authors need to look into.

1. To begin with, the background in the abstract refers to MRSA being a concern for infection control in low-income settings. This study did not focus on infection control; therefore, this aspect may not warrant inclusion. This was a laboratory-based retrospective study with no focus on infection control, so this may need to be removed from the abstract. Additionally, the issue of MRSA is not limited to low-incomesettings. This is a global issue, so the authors should consider framing it in that context.

2. In the background, it would help put the study into context if the authors state what is empirically prescribed in the case of MRSA. I think this is missing in both the abstract and the main document. Including this will strengthen the current draft's argument regarding why exploring resistance to MRSA matters in the context of empirical prescription

3. In the results section, the age band categories need to be rewritten for clarity, likely as: 0–10, 11–17, 18–24, 25–44, 45–59, and 60 and above.

4. In line 212 the sentence that includes overwhelming majority, the words overwelming majority does not read well for a scientific paper, consider replacing with more appropriate like a “larger proportion”

5. It will be worthwhile to state what constituted others for the specimens explored in the study, this can be put in blackets

6. Coming to the discussion: The authors have included results in this section. Results should not be repeated in the discussion. Instead, the authors must make inferences from the results. What is their significance? How do the results compare with other studies? They should also discuss the results in terms of their implications within the current clinical context.

7. The public health context should not simply restate the findings; thus, the raw results must be entirely removed from the discussion section of the manuscript.

8. Lastly, I would like to suggest that the authors consider including a recommendation on Linezolid to be prioritised in empirical treatment of MRSA.

**********

what does this mean?). If published, this will include your full peer review and any attached files.

**Do you want your identity to be public for this peer review?**  If you choose “no”, your identity will remain anonymous but your review may still be made public.

For information about this choice, including consent withdrawal, please see our Privacy Policy

Reviewer #1: No

Reviewer #2: **Yes:**  Patrick Saili Lungu

**********

[NOTE: If reviewer comments were submitted as an attachment file, they will be attached to this email and accessible via the submission site. Please log into your account, locate the manuscript record, and check for the action link "View Attachments". If this link does not appear, there are no attachment files.]

**Figure Resubmissions:**

# Author comment: Author response to Decision Letter 1

**DOI:** 10.1371/journal.pgph.0007348.r002

5 Jul 2026

Attachment Submitted filename: Response to Reviewers.docx

# Peer review: Decision Letter 1

**DOI:** 10.1371/journal.pgph.0007348.r003

**Authors:** Monde Muyoyeta

3 Aug 2026

PGPH-D-26-01568R1

High Prevalence and Multidrug Resistance of Methicillin-Resistant Staphylococcus aureus in a Provincial Referral Hospital in Zambia: A 5-Year Retrospective Study

PLOS Global Public Health

Dear Dr. Chisompola,

Thank you for submitting your manuscript to PLOS Global Public Health. After careful consideration, we feel that it has merit but does not fully meet PLOS Global Public Health’s publication criteria as it currently stands. Therefore, we invite you to submit a revised version of the manuscript that addresses the points raised during the review process.

Please submit your revised manuscript by Sep 02 2026 11:59PM. If you will need more time than this to complete your revisions, please reply to this message or contact the journal office at globalpubhealth@plos.org. When you're ready to submit your revision, log on to https://www.editorialmanager.com/pgph/ and select the 'Submissions Needing Revision' folder to locate your manuscript file.

Please include the following items when submitting your revised manuscript:

- A letter that responds to each point raised by the editor and reviewer(s). You should upload this letter as a separate file labeled 'Response to Reviewers'.
- A marked-up copy of your manuscript that highlights changes made to the original version. You should upload this as a separate file labeled 'Revised Manuscript with Track Changes'.
- An unmarked version of your revised paper without tracked changes. You should upload this as a separate file labeled 'Manuscript'.

Guidelines for resubmitting your figure files are available below the reviewer comments at the end of this letter.

As the corresponding author, your ORCID iD is verified in the submission system and will appear in the published article. PLOS supports the use of ORCID, and we encourage all coauthors to register for an ORCID iD and use it as well. Please encourage your coauthors to verify their ORCID iD within the submission system before final acceptance, as unverified ORCID iDs will not appear in the published article. *Only*  the individual author can complete the verification step; PLOS staff *cannot*  verify ORCID iDs on behalf of authors.

We look forward to receiving your revised manuscript.

Kind regards,

Monde Muyoyeta, MBCHB, Ph.D

Academic Editor

PLOS Global Public Health

Journal Requirements:

If the reviewer comments include a recommendation to cite specific previously published works, please review and evaluate these publications to determine whether they are relevant and should be cited. There is no requirement to cite these works unless the editor has indicated otherwise.

Please review your reference list to ensure that it is complete and correct. If you have cited papers that have been retracted, please include the rationale for doing so in the manuscript text, or remove these references and replace them with relevant current references. Any changes to the reference list should be mentioned in the rebuttal letter that accompanies your revised manuscript. If you need to cite a retracted article, indicate the article’s retracted status in the References list and also include a citation and full reference for the retraction notice.

Additional Editor Comments (if provided):

[Note: HTML markup is below. Please do not edit.]

Reviewers' comments:

Reviewer's Responses to Questions

**Comments to the Author**

Reviewer #1: All comments have been addressed

**********

publication criteria?

Reviewer #1: Yes

**********

3. Has the statistical analysis been performed appropriately and rigorously?

Reviewer #1: Yes

**********

4. Have the authors made all data underlying the findings in their manuscript fully available (please refer to the Data Availability Statement at the start of the manuscript PDF file)?

The PLOS Data policy

Reviewer #1: Yes

**********

5. Is the manuscript presented in an intelligible fashion and written in standard English?

Reviewer #1: Yes

**********

Reviewer #1: The manuscript has improved considerably and is much clearer than the original submission. The statistical analysis is appropriate for the study design. The authors have clearly described the analytical methods, justified the regression model, and reported the results in a way that is easy to follow. I also appreciate that the limitations, particularly the lack of D-zone testing and the variation in antimicrobial susceptibility testing across isolates, are now acknowledged.

I have only a few minor comments:

The conclusions should be slightly more cautious. Since this is a single-centre retrospective study, recommendations regarding empirical antibiotic use should be framed within the context of the study setting.

Please verify whether Mansa General Hospital should be described as a tertiary hospital or a provincial referral hospital and ensure this is consistent throughout the manuscript.

It would be useful to briefly acknowledge in the discussion that cefoxitin disk diffusion is a phenotypic surrogate for MRSA detection and that molecular confirmation was not available.

A final proofread is recommended, as there are a few minor grammatical and typographical errors that remain.

Overall, this is a well-conducted study that provides useful information on the burden of MRSA and antimicrobial resistance in Zambia. The findings are relevant to antimicrobial stewardship and infection prevention efforts, particularly in resource-limited settings. Subject to these minor revisions, I believe the manuscript is suitable for publication.

**********

what does this mean?). If published, this will include your full peer review and any attached files.

**Do you want your identity to be public for this peer review?**  If you choose “no”, your identity will remain anonymous but your review may still be made public.

For information about this choice, including consent withdrawal, please see our Privacy Policy

Reviewer #1: No

**********

[NOTE: If reviewer comments were submitted as an attachment file, they will be attached to this email and accessible via the submission site. Please log into your account, locate the manuscript record, and check for the action link "View Attachments". If this link does not appear, there are no attachment files.]

# Author comment: Author response to Decision Letter 2

**DOI:** 10.1371/journal.pgph.0007348.r004

4 Aug 2026

Attachment Submitted filename: Response_to_Reviewers_auresp_2.docx

# Editor report: Decision Letter 2

**DOI:** 10.1371/journal.pgph.0007348.r005

**Authors:** Monde Muyoyeta

7 Sep 2026

High Prevalence and Multidrug Resistance of Methicillin-Resistant Staphylococcus aureus at a Provincial Referral Hospital in Zambia: A 5-Year Retrospective Study

PGPH-D-26-01568R2

Dear Mr Chisompola,

We are pleased to inform you that your manuscript 'High Prevalence and Multidrug Resistance of Methicillin-Resistant Staphylococcus aureus at a Provincial Referral Hospital in Zambia: A 5-Year Retrospective Study' has been provisionally accepted for publication in PLOS Global Public Health.

Before your manuscript can be formally accepted you will need to complete some formatting changes, which you will receive in a follow up email. A member of our team will be in touch with a set of requests.

Please note that your manuscript will not be scheduled for publication until you have made the required changes, so a swift response is appreciated.

IMPORTANT: The editorial review process is now complete. PLOS will only permit corrections to spelling, formatting or significant scientific errors from this point onwards. Requests for major changes, or any which affect the scientific understanding of your work, will cause delays to the publication date of your manuscript.

If your institution or institutions have a press office, please notify them about your upcoming paper to help maximize its impact. If they'll be preparing press materials, please inform our press team as soon as possible -- no later than 48 hours after receiving the formal acceptance. Your manuscript will remain under strict press embargo until 2 pm Eastern Time on the date of publication. For more information, please contact globalpubhealth@plos.org.

Thank you again for supporting Open Access publishing; we are looking forward to publishing your work in PLOS Global Public Health.

Best regards,

Monde Muyoyeta, MBCHB, Ph.D

Academic Editor

PLOS Global Public Health

***********************************************************

Reviewer Comments (if any, and for reference):
